# Clinical audit and quality improvement: rivals, partners, or one and the same?

**DOI:** 10.3389/frhs.2026.1768450

**Published:** 2026-05-29

**Authors:** Nicola Davey, Heather Shearer, Dionne Matthew, Jamie Smyth, Stephanie A. Meddick-Dyson

**Affiliations:** 1 Quality Improvement Clinic, Southampton, United Kingdom; 2 School of Medicine, University of Dundee, Dundee, United Kingdom; 3 School of Public Health, Imperial College London, London, United Kingdom; 4 Hull York Medical School, University of Hull, Hull, United Kingdom

**Keywords:** clinical audit, improvement methodology, improvement science, improvement science education, quality improvement

## Abstract

Clinical audit and quality improvement are both central to improving healthcare quality; however, there is often a lack of conceptual clarity about their similarities and differences. They are frequently misunderstood, conflated, or applied in ways that do not match their primary purpose. In the United Kingdom, this lack of conceptual clarity is evident across clinical practice, healthcare improvement literature, and professional curricula. The misapplication of methods may contribute to inefficient use of resources and missed opportunities to improve experiences and outcomes for patients, families, and staff. This practice-informed perspective article clarifies the distinct purposes and roles of clinical audit and quality improvement before demonstrating how they can be used in practice. Drawing on published literature, professional guidance, and the authors’ practical experience, we examine the key similarities, differences, and common points of confusion between clinical audit and quality improvement. We highlight recurring challenges faced by UK healthcare professionals when selecting and applying these approaches in real-world settings, as a call for international reflection. We present a decision aid to support intentional and effective method selection and purposeful transition between approaches. By providing clarity on how clinical audit and quality improvement are distinct yet complementary, this article aims to support more rigorous, contextually appropriate, and impactful improvement efforts across all healthcare systems.

## Introduction

**Figure 1:**
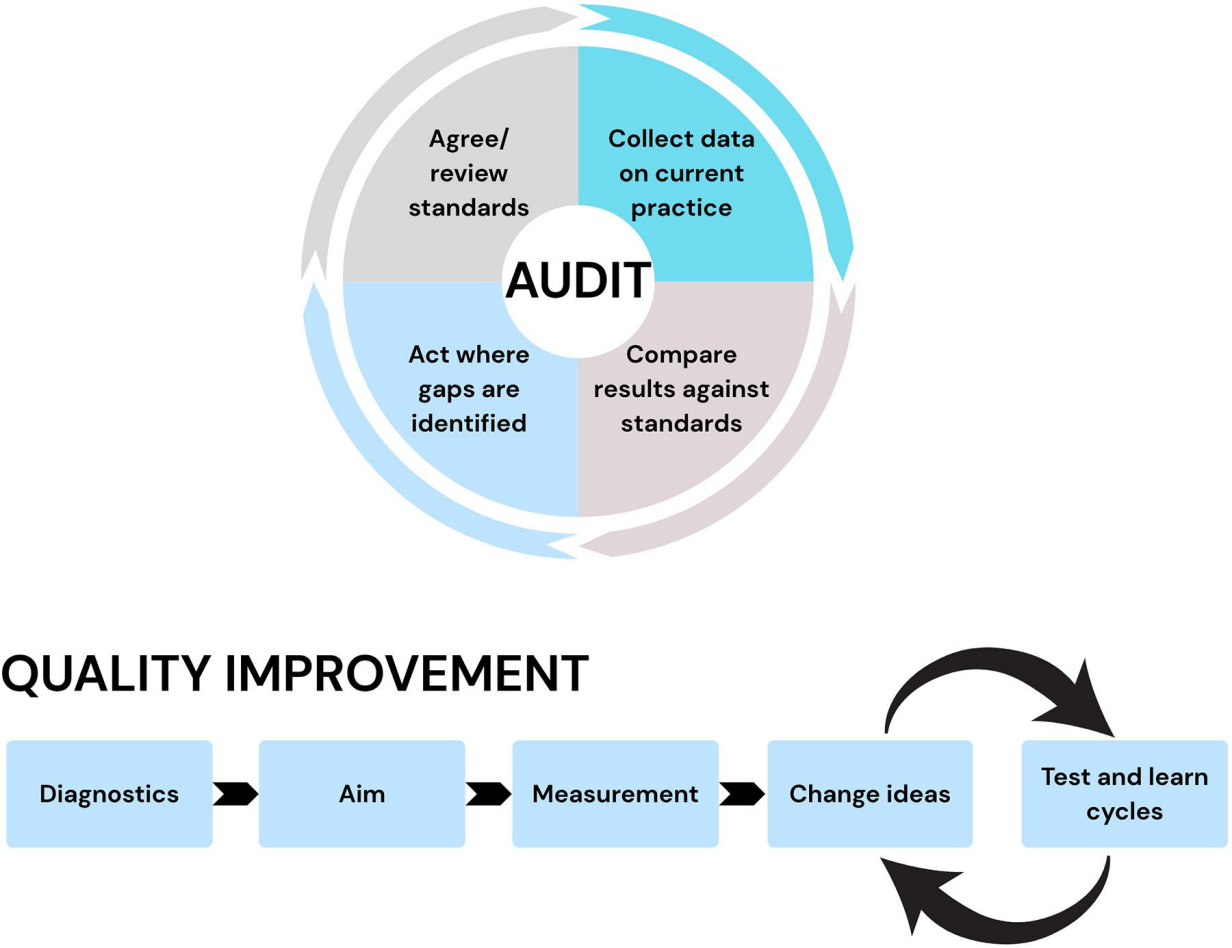
Generic illustration of audit and quality improvement approaches.

Clinical audit and quality improvement (QI) are both widely used approaches to improving healthcare; however, they serve distinct primary purposes (1). Broadly, audit is a method for assessing care against a standard. The term audit carries different meanings across sectors. However, in this article, we use the term audit to refer specifically to clinical audit, that is, audit conducted to assess care within a healthcare setting (2). Clinical audit is one way healthcare organisations can assure themselves, commissioners, regulators, and patients that key standards of care are being met (3). While definitions of audit have evolved, the core steps remain the same: agreeing or reviewing standards, collecting data on current practice, comparing results against standards, and taking actions where gaps are identified. While definitions do extend to sustainability of improvements, this aspect is often omitted in practice (4, 5). In contrast, QI is best seen as a set of principles and methods for making and studying change (1, 6). It is a systematic, applied approach to addressing complex problems through iterative testing, continuous measurement, and engagement with key stakeholders (7). Common methodologies include Plan–Do–Study–Act (PDSA) cycles within the Model for Improvement (8), and Lean methodology using Plan–Do–Check–Act (PDCA) cycles (9). A range of diagnostic tools, including fishbone diagrams, process mapping, driver diagrams, and statistical process control, offer complementary strengths, enabling flexible application depending on the problem (10–12). Fundamentally, clinical audit identifies performance gaps by measuring actual care against desired outcomes, whereas QI provides the methodological framework for designing, testing, and implementing context-sensitive interventions that account for local environmental factors and pre-existing conditions (13, 14). Figure 1 generically illustrates these two approaches and their components.

As described by Backhouse and Ogunlayi, audit and QI are two of several approaches to improving care within the UK healthcare setting (1). Poor understanding of how these approaches differ can create mismatched expectations among teams seeking improvement and reduce fidelity to core methodological principles, thereby limiting the chance of success (1). Across more than 60 years of collective experience leading, teaching, and managing improvement initiatives from ward to organisational level within UK healthcare, we have repeatedly observed the practical consequences of this conflation. For early-career healthcare professionals learning to contribute to the future of healthcare improvement, the implications are far-reaching. Although direct empirical research on this specific problem appears limited, professional guidance and expert commentary have long highlighted how a lack of clarity can create challenges in teaching, learning, and real-world improvement practice. A 2018 report by the Royal College of Physicians (RCP), commissioned by the Healthcare Quality Improvement Partnership, noted the “evident great confusion between the terms clinical audit and quality improvement” (15). This confusion, together with a lack of understanding of the basics of these methodologies among senior physicians, was highlighted as a limitation on care improvement and medical training (15). An RCP blog reflects on the common scenario of a junior doctor attempting to complete a misplaced audit within the timeframe of a short clinical rotation, often resulting in the burden of data collection without real consideration for change (16). This article therefore offers a practice-informed discussion of the conflation between audit and QI and explains why distinguishing between the two is important for teaching, learning, and real-world improvement practice. We provide a clear conceptual and practical comparison of audit and QI and propose a pragmatic decision aid to support all healthcare professionals in making more informed methodological choices.

## Conflation problem

For early-career healthcare professionals, the implications can be significant, affecting their learning and sense of agency, the organisation's use of time and resources, and potentially the quality of care delivered to patients and families. In our experience, ward-level improvement work led by early-career healthcare professionals is particularly vulnerable to confusion between audit and QI. As described in the RCP blog (16) and as we see in practice, early-career professionals are required to engage in improvement activities while being directed to use methods that do not align with their resources or intent. At an organisational level, audit plays an important assurance role and often involves carefully managed large data sets. However, the aim of early-career professionals is often to make meaningful, focused changes within the scope of their rotation duration and sphere of influence. Yet we see advice being given to collect vast amounts of data before they can act. In our experience, a substantial proportion of improvement project time is devoted to retrospective data collection. The data collected often come from existing databases or historical stores, where access may not be easy and timeliness is not guaranteed (17). Such data may also offer limited value in understanding the local problem under consideration (17). We hear audit being selected because it appears more familiar or feasible; however, this can also reflect limited knowledge of QI methods and their potential advantages for local care improvement (18). This mismatch between the intent and method can make meaningful change harder to achieve within the timeframe of rotations (17). In comparison, a sharp focus on planning for data collection within QI can reveal small amounts of data that are easier to access and generate valuable learning. Unfortunately, these smaller yet more impactful steps can be undervalued when they are not understood. Dedicated non-clinical time for healthcare professionals to develop knowledge and practical experience in improvement methods is often limited. From our interactions with these professionals, poor guidance appears to contribute to repeated experiences of poorly executed improvement work and disappointing outcomes. Over time, this may reduce professionals’ sense of agency and confidence in their ability to improve care. Evidence suggests this can negatively impact workforce motivation (19).

Published papers, training materials, and professional curricula may unintentionally reinforce this lack of distinction. In our experience within the United Kingdom, postgraduate medical curricula frequently perpetuate the conflation of audit and QI. Despite the positive shift towards integrating QI into medical education (20), guidance can present the two approaches as seemingly interchangeable. One manifestation of this confusion is the substitution of the PDSA cycle for the audit cycle, or vice versa, as though they serve the same methodological function (5, 21–23). As discussed previously, the audit cycle is structured around assessing performance against an agreed standard, whereas the PDSA cycle is structured around testing and refining change in practice. Blurring the logic of one with the operational framework of the other risks weakening the value and rigour of both. Competency-based portfolios may inadvertently incentivise volume over methodological rigour by awarding credit for the rapid completion of superficial audit cycles. These educational frameworks can actively discourage the sustained, longitudinal engagement required to genuinely improve complex clinical systems (24–26). Peer-reviewed healthcare literature adds to this methodological confusion. Observational audits are frequently misclassified as QI initiatives, while complex, iterative system redesign efforts are occasionally minimised under the label of routine audits.

In a setting predisposed to focusing on positive findings, healthcare professionals are already vulnerable to overlooking learning from null findings (27). Misunderstandings surrounding audit and QI can exacerbate this issue by obscuring the different kinds of learning they are designed to support. In audit, null findings warrant celebration of high-quality care and redirection of energy to areas that need improvement. In QI, unsuccessful tests of change are still valuable. They generate learning, refine ideas, prevent the repetition of ineffective interventions, and prioritise problems within reach of the professionals undertaking this activity.

Ultimately, we argue that this conflation is not just semantic. Audit and QI are shaped by distinct primary logics and differing expectations about the purpose of measurement. When healthcare systems fail to respect these distinct logics, they risk misallocating resources, disrupting clinical governance, and ultimately weakening the impact of both methodologies.

## Moving towards clarity

To support greater clarity in practice, we provide a conceptual and practical comparison of audit and QI to build on Figure 1 (Table 1).

**Table 1:** Conceptual and practical comparison of clinical audit and quality improvement.

Feature	Clinical audit	Quality improvement
Primary purpose	Assesses care against agreed standards. Identifies and aims to close performance gaps	Improves processes and outcomes through iterative testing and adaptation
Methodological approach	Measures current practice against explicit standards, usually with at least one repeat measurement after change	Scientific change method incorporating diagnostics, design, measurement, and repeated small-scale tests of change
Measurement approach	Compares past performance with an agreed standard	Utilises small amounts of real-time data to establish baseline variation, thereby allowing determination of any statistically significant change
Primary output	Evidence of compliance with standards and areas requiring improvement—usually, interval quantitative data	New learning and understanding of processes in a given context; improved processes and outcomes; and multiple, iterative data
Typical data volume	May involve larger samples or whole populations using routine records or case-note review	Often uses ‘just enough’ data collected frequently enough to support learning and decision-making
Influence on change	Influence may be limited for the individual collecting and comparing data. Wider influence depends on the involvement of others when implementing change^a^	Considers influence early by identifying who needs to be involved to make change possible Can spread beyond the team and organisation through formal or informal communities of practice
Ethical and governance considerations	Emphasises privacy, confidentiality, and appropriate governance in the use of patient data—ethics requirements generally less onerous for audit than for research	Emphasises safe testing of change, stakeholder involvement, and balancing measures to reduce unintended harm
Stakeholder collaboration	Collaboration may become more prominent once gaps are identified and action is required	Collaboration with relevant stakeholders is typically encouraged from the outset
Individual vs. team working	Data collection and analysis are often led individually, although change usually requires wider involvement	Usually involves team-based working to understand, test, and embed change
Typical timescale	Each audit cycle typically takes between 4 and 12 months, with a repeat cycle taking up to 24 months	Small projects are typically completed in 4–6 months, including multiple test cycles. Larger projects typically take 9–12 months
Time and workload	Can be time-intensive, especially when based on retrospective data collection or manual case review	Ideally designed to be agile and time lite, with focused effort and rapid feedback. Efforts that do not lead to improvement are adjusted or abandoned
Assessment of change ideas	Can show whether performance improves after the change. Less able to attribute improvement to the change made	The use of rigorous change cycles enables individual change ideas to be assessed for their relative contribution
How learning is shared	Findings commonly contribute to governance and assurance activities and are often reported after completion	Learning is shared throughout the process to inform next steps, promote engagement and buy-in from stakeholders, and spread success
Where it is particularly useful	To raise standards across a country or region, e.g., National Clinical Audits	To improve a small element of a local service; where local problems are complex and require context-sensitive change

a National audits generally have a wider influence.

**Figure 2:**
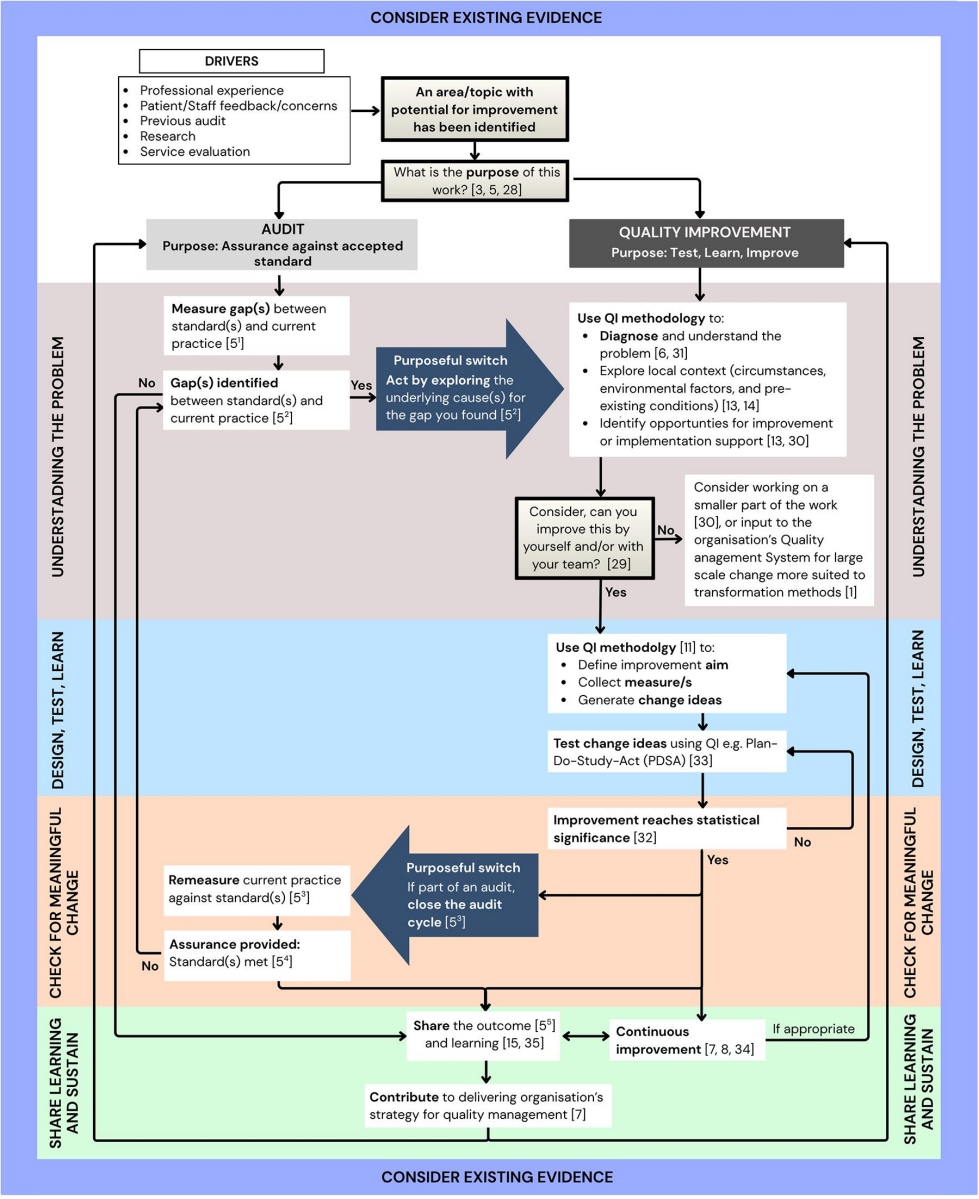
Decision aid between audit and quality improvement methods. 5^1–5^ highlight specific relevant points in Best Practice in Clinical Audit (HQIP 2020) (5): 5^1^: Measuring performance, point 1; 5^2^: Implementing change, point 1; 5^3^: Implementing change, point 2; 5^4^: Sustaining improvement, points 1–3; 5^5^: Sustaining improvement, points 3–4.

Importantly, although distinct, audit and QI share a common goal of improving healthcare quality, making them complementary rather than competing approaches. Drawing on the literature and the practical experience of authors, Figure 2 presents a decision aid intended to support method selection when planning and progressing through improvement work (1, 3, 5–8, 11, 13, 14, 28–35). It illustrates that when the objective of a project shifts from ensuring compliance to driving change, practitioners must purposefully transition to a QI approach. The key is to identify which approach is being used, for what purpose, and at what stage in the work. It also emphasises the importance of exploring existing evidence throughout any improvement process (13). This may involve establishing the standards care is measured against or the desired outcomes (5), considering existing knowledge of the causes of the problem in focus, and seeking ideas for change (13). For example, there may be existing evidence on barriers and facilitators faced in a similar context or in providing a similar intervention. Reviewing prior interventions, both successful and unsuccessful, avoids unnecessary repeated exploration and resource waste and directs efforts towards the development of new change(s), or implementation of existing initiatives within a new context (13).

## Wider context and looking to the future

We have outlined how a lack of clarity between audit and QI can cause problems in teaching, learning, and real-world improvement practice. More broadly, many healthcare organisations are adopting quality management systems (QMSs) to deliver high-quality services (36). A QMS can be understood as a dynamic and coordinated approach to planning, improving, controlling, and assuring high-quality care (37). Within such systems, both audit and QI have important roles, but only when used appropriately and with sufficient conceptual clarity. QI is often positioned as a core pillar within a QMS, whereas audit is seen as a tool for quality assurance (37). Figure 2 illustrates how these activities can be integrated into an organisational QMS in practical terms.

Although some publications and guidance acknowledge differences between audit and QI, the majority we see reinforce confusion by conflating the two approaches (20–23). Recent reflections in a *British Medical Journal* blog have argued that audit and QI can be integrated by drawing on approaches such as Lean methodology and the Model for Improvement (38). Where confusion between audit and QI persists, integration may prematurely deprioritise the fidelity of each approach in its practice. Table 1 is intended to provide the clarity needed before meaningful integration of the audit and QI, provoking more deliberate consideration of how each approach contributes to improving care. This article also provides a decision aid to illustrate how this integration may work in practice whilst maintaining clarity between the two methods (Figure 2). We hope this clarity will support those responsible for curriculum design, training, and assessment development, enabling the next generation of improvers to be better prepared to contribute to improvement and, in the longer term, to quality management systems within their organisations.

We have drawn on problems we observe among early-career healthcare professionals in the United Kingdom who are attempting to improve care for patients and families. However, the decision aid is designed to guide all improvement practitioners. It is derived from the international evidence base and has been co-designed by improvement professionals with many years of practical experience. Although the decision aid has undergone multiple iterations, its usability has not yet been formally evaluated, and further refinement may be needed. In keeping with QI methods, it may need to be adapted to suit different healthcare settings and systems, both within the United Kingdom and worldwide. Limited by the lack of formal consensus methods and empirical evidence, there may also be missing links beyond those currently identified, or flaws in those that have been identified.

Although this article uses the case of early-career healthcare professionals to illustrate the impacts of conflating audit and QI, the outlined differences between audit and QI are universal. We anticipate that the benefits of the tools presented here will extend beyond UK healthcare and the early-career healthcare professionals who inspired them. We hope this paper supports ongoing international dialogue and helps reduce poorly implemented improvement efforts that consume significant time from an already stretched workforce while delivering limited or no benefit.

## Conclusion

Audit and QI are related but distinct approaches to improving care, each with its own primary purposes and strengths. When they are conflated, avoidable problems can arise in education, improvement practice, and wider quality management. Meaningful integration is only possible following conceptual clarity between audit and QI, as well as how they can complement one another to strengthen efforts to improve the quality of healthcare delivery. This article provides that necessary clarity, promoting coherent, intentional use of both approaches.

## Data Availability

The original contributions presented in the study are included in the article further inquiries can be directed to the corresponding author.
